# Dissecting Brain Networks Underlying Alcohol Binge Drinking Using a Systems Genomics Approach

**DOI:** 10.1007/s12035-018-1252-0

**Published:** 2018-07-30

**Authors:** Laura B. Ferguson, Lingling Zhang, Daniel Kircher, Shi Wang, R. Dayne Mayfield, John C. Crabbe, Richard A. Morrisett, R. Adron Harris, Igor Ponomarev

**Affiliations:** 1 The Waggoner Center for Alcohol and Addiction Research, The University of Texas at Austin, Austin, TX USA; 2 The Institute for Neuroscience, The University of Texas at Austin, Austin, TX USA; 3 Portland Alcohol Research Center, VA Portland Health Care System, Portland, OR USA; 4 Department of Behavioral Neuroscience, Oregon Health & Science University, Portland, OR USA

**Keywords:** Gene expression, Binge drinking, Transcriptome, Gene networks, HDID mice

## Abstract

Alcohol use disorder (AUD) is a complex psychiatric disorder with strong genetic and environmental risk factors. We studied the molecular perturbations underlying risky drinking behavior by measuring transcriptome changes across the neurocircuitry of addiction in a genetic mouse model of binge drinking. Sixteen generations of selective breeding for high blood alcohol levels after a binge drinking session produced global changes in brain gene expression in alcohol-naïve High Drinking in the Dark (HDID-1) mice. Using gene expression profiles to generate circuit-level hypotheses, we developed a systems approach that integrated regulation of gene coexpression networks across multiple brain regions, neuron-specific transcriptional signatures, and knowledgebase analytics. Whole-cell, voltage-clamp recordings from nucleus accumbens shell neurons projecting to the ventral tegmental area showed differential ethanol-induced plasticity in HDID-1 and control mice and provided support for one of the hypotheses. There were similarities in gene networks between HDID-1 mouse brains and postmortem brains of human alcoholics, suggesting that some gene expression patterns associated with high alcohol consumption are conserved across species. This study demonstrated the value of gene networks for data integration across biological modalities and species to study mechanisms of disease.

**Electronic supplementary material:**

The online version of this article (10.1007/s12035-018-1252-0) contains supplementary material, which is available to authorized users.

## Introduction

Binge drinking is a dangerous pattern of alcohol (ethanol) drinking that produces blood alcohol levels (BALs) of 0.08 g/dL or higher [1]. Binge drinking increases the risk of developing alcohol use disorder (AUD) and poses serious health and social problems for individuals and the community (e.g., alcohol-related cancers, unintended pregnancies, HIV infections and other sexually transmitted diseases, violence, and injury or death, especially from traffic accidents) [2–4]. Investigating the biological basis of binge drinking will help guide the development or repurposing of suitable interventions to reduce this hazardous behavior. Similar to other complex psychiatric phenotypes, there are both genetic and environmental components that drive excessive alcohol consumption. High Drinking in the Dark (HDID-1) mice are selectively bred from the HS/Npt stock to drink consistently to BALs of 0.1 g/dL or greater in the DID behavioral test, a procedure used to model binge-like drinking in which ethanol is available for a limited time during the circadian dark cycle [5, 6]. A review by Crabbe and colleagues provides detailed discussion of DID and other preclinical models of binge drinking [7].

Selective breeding to produce the HDID line capitalized on the genetic heterogeneity in the founding population, resulting in fixation of alleles responsible for excessive drinking. These genetic changes were associated with expression changes in the ventral striatum, one of the brain areas involved in regulating alcohol consumption [8]. Gene expression is a sensitive measure of cell function, and neuronal and glial cells work together to translate molecular signals into the expression of complex phenotypes, such as normal behavior or brain pathology. A fundamental challenge in neuroscience is to understand how perturbation-induced molecular changes in different cell types are integrated to change activity of the neural networks that drive behavior.

In this study, we investigated the effects of genetic selection on genome-wide gene expression in seven brain areas implicated in regulating alcohol consumption [prefrontal cortex (PFC), nucleus accumbens core (AcbC), nucleus accumbens shell (AcbSh), bed nucleus of the stria terminalis (BNST), basolateral amygdala (BLA), central nucleus of the amygdala (CeA), and ventral tegmental area (VTA)]. We found many changes in brain gene expression between ethanol-naïve HDID-1 and HS/Npt mice, suggesting that some of these changes drive the genotype differences in ethanol intake and BALs. The seven brain regions studied here are highly interconnected to form a neural network that drives motivated behaviors, allowing us to examine integrated gene expression profiles across the neurocircuit. A gene network-based systems approach was used to partition the transcriptional variability across the brain regions into several gene modules with correlated expression. Selection-dependent, cell type-specific gene networks were identified, many of which were conserved in human alcoholics. Based on coregulation of gene networks across multiple brain regions, specific neural connections were also identified that may be altered in HDID-1 mice.

## Results

### Genetic Selection for Ethanol Binge Drinking Alters Brain Gene Expression

**Fig. 1:**
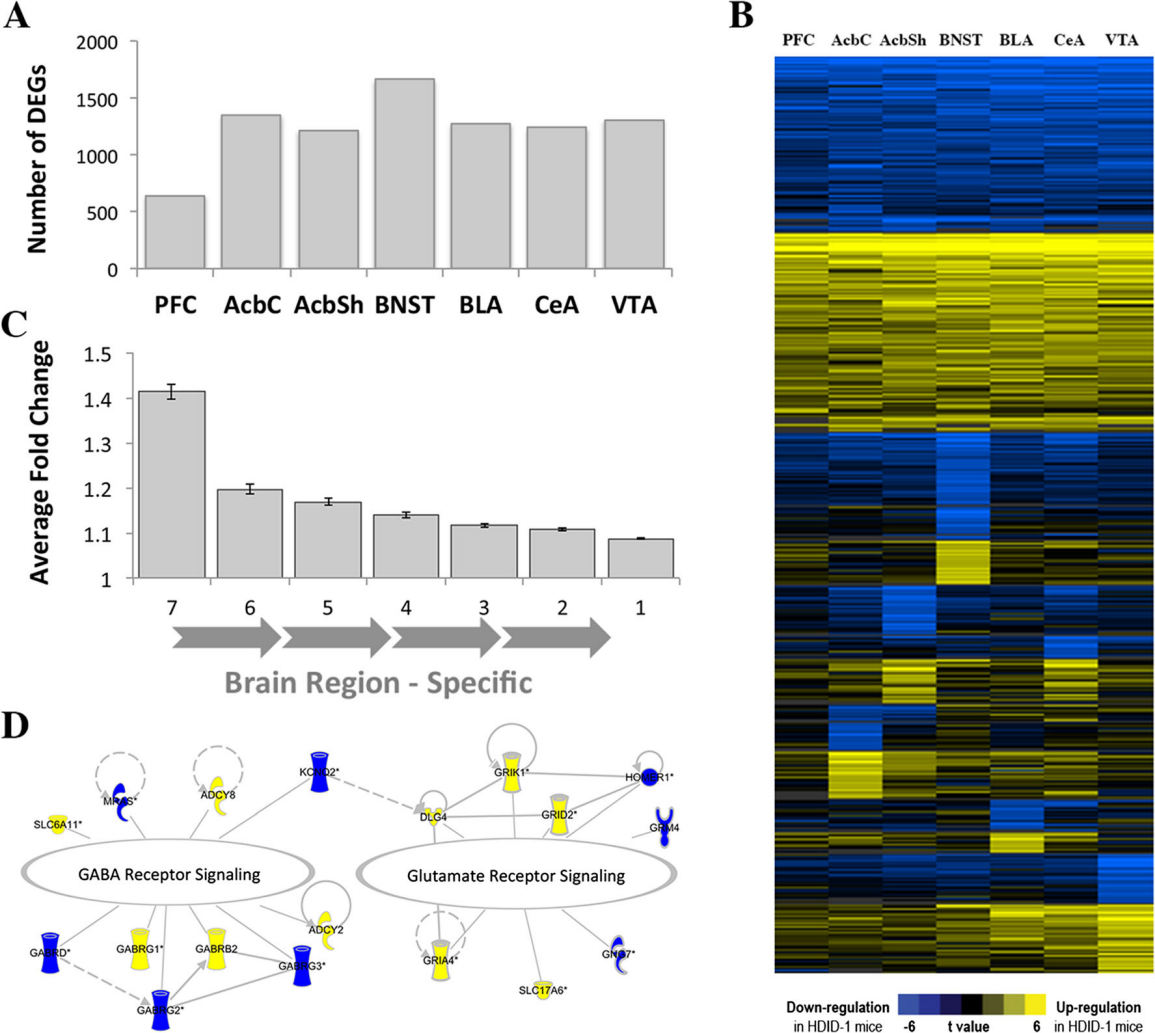
Effects of genetic selection for high blood alcohol levels after binge drinking on brain gene expression. Gene expression for seven brain regions was measured using microarrays. Expression levels for all detected genes were compared in High Drinking in the Dark (HDID-1) and HS/Npt control mice by empirical Bayes-moderated *t* statistics using the Bioconductor limma package version 3.24.15 in R (*N* = 11-12 mice/genotype). All comparisons combined results from up-regulated (HDID-1 > HS) and down-regulated (HDID-1 < HS) genes. Numbers of DEGs between genotypes in each brain region are shown in (**a**) and were significantly greater than those expected by chance in all brain regions except for the PFC. To visualize brain region-specific regulation, *t* values for top statistically significant genes (*p* < 0.001) were clustered across brain regions using K-means clustering (Cluster 3.0 and Java TreeView free software) (**b**). The average (± SEM) fold changes of DEGs are plotted according to the number of brain regions in which the gene is differentially expressed (**c**). About 40% of DEGs in most (6–7) brain regions had moderate to high fold changes (≥ 1.2), while brain region-specific changes (regulated in 1–2 brain regions) were smaller on the average. Ingenuity Pathway Analysis revealed changes in glutamatergic and GABAergic signaling pathways in the AcbSh (**d**) (see text for details). Solid/dashed lines between molecules represent relationships between the molecules as supported by the literature. PFC = prefrontal cortex, AcbC = nucleus accumbens core, AcbSh = nucleus accumbens shell, BNST = bed nucleus of the stria terminalis, BLA = basolateral amygdala, CeA = central nucleus of the amygdala, VTA = ventral tegmental area, DEGs = differentially expressed genes

Gene expression analysis identified 17,679 microarray probes (corresponding to 10,853 unique genes) that passed the detection filter in at least one out of seven brain regions, with ~ 13,000 to ~ 16,000 probes expressed in any individual brain region. In a plot of the first two principal components, separation of the expression data was based on brain region, validating our previous finding that brain region and cell type are primary contributors to variance in gene expression (Fig. S1 in Online Resource 1), e.g., [9, 10]. Interestingly, the PFC samples do not congregate as closely as samples from other brain areas, presumably attributable to biological variability, because care was taken to limit technical variability. This may, at least in part, explain the lower numbers of DEGs in this brain region. Differential expression analysis showed that genetic selection for high BALs produced global changes in brain gene expression, with 4,122 genes differing between HDID-1 and HS/Npt mice in at least one brain region (Table 1; full differential expression results are reported in Table S1 in Online Resource 2). The number of differentially expressed genes (DEGs) was statistically greater than expected by chance in six out of seven brain regions (Fig. 1a). Approximately one-third of the DEGs were regulated across most brain regions, while the rest showed region-specific regulation (Fig. 1b). One-third of these were in the extended amygdala (BNST, AcbSh, and CeA), indicating the importance of this structure in binge drinking. Average fold change was greater for those DEGs present across multiple regions. Smaller fold change for region-specific DEGs may be attributed to a “dilution” effect, suggesting that these genes are regulated in specific cellular (potentially neuronal) subpopulations (Fig. 1c). There was a highly significant overlap between the DEGs in the nucleus accumbens identified by our study and an independent study that characterized gene expression in the ventral striatum (a region containing the nucleus accumbens) from ethanol-naïve male HDID-1 and HS/Npt mice [10] (hypergeometric *p* < 5E-28), providing validation for our gene expression platform.

**Table 1:** Differentially expressed genes between HDID-1 and HS/Npt mice

Gene symbol	Gene name	Fold change (HDID vs HS/Npt controls)	Description
Tomm22	Translocase of outer mitochondrial membrane 22	2.27–2.71	The encoded protein is an integral membrane protein of the mitochondrial outer membrane. It forms a complex with several other proteins to import cytosolic preproteins into the mitochondrion
Usp29	Ubiquitin specific peptidase 29	1.52–2.14	The encoded protein is a protease that cleaves ubiquitin from proteins and other molecules. Ubiquitination is involved in protein degradation and trafficking
Hmgn2	High mobility group nucleosomal binding domain 2	1.53–1.83	The encoded protein binds nucleosomal DNA and is associated with transcriptionally active chromatin. Along with a similar protein, HMGN1, the encoded protein may help maintain an open chromatin configuration around transcribable genes. The protein has also been found to have antimicrobial activity against bacteria, viruses, and fungi
Pnmal1	Paraneoplastic Ma antigen family-like 1	1.20–1.39	An important paralog of this gene is Zinc Finger, CCHC Domain Containing 12 (ZCCHC12), which codes for a transcriptional coactivator in the bone morphogenetic protein (BMP)-signaling pathway
Vsnl1	Visinin-like 1	1.23–1.49	A member of the visinin/recoverin subfamily of neuronal calcium sensor proteins. The encoded protein associates with membranes in a calcium-dependent manner and modulates intracellular signaling pathways of the central nervous system by directly or indirectly regulating the activity of adenylyl cyclase
Atf4	Activating transcription factor 4	1.17–1.55	Encodes a transcription factor that belongs to a family of DNA-binding proteins that includes the AP-1 family of transcription factors, cAMP response element binding proteins (CREBs) and CREB-like proteins. It binds to a Tax-responsive enhancer element in the long terminal repeat of HTLV-I. Regulates the induction of DDIT3/CHOP and asparagine synthetase (ASNS) in response to endoplasmic reticulum (ER) stress. In concert with DDIT3/CHOP, activates the transcription of TRIB3 and promotes ER stress-induced neuronal apoptosis by regulating the transcriptional induction of BBC3/PUMA. Activates transcription of SIRT4. Regulates the circadian expression of the core clock component PER2 and the serotonin transporter SLC6A4. Binds in a circadian time-dependent manner to the cAMP response elements (CRE) in the SLC6A4 and PER2 promoters and periodically activates the transcription of these genes. During ER stress response, activates the transcription of NLRP1, possibly in concert with other factors (PubMed: 26086088)
Arhgap35	Rho GTPase activating protein 35	− 1.45 to − 1.85	The encoded protein inhibits glucocorticoid receptor transcription
Trappc13	Trafficking protein particle complex 13	− 1.29 to − 1.45	May play a role in vesicular transport from ER to Golgi

Differential expression analysis was conducted within each brain region using empirical Bayes moderated *t* statistics in the Bioconductor limma package version 3.24.15 in R to compare male, ethanol-naïve HDID-1 with HS/Npt mice (*N* = 11-12 mice/genotype). Eight genes were differentially expressed between genotypes across all seven brain regions after correction for multiple tests. Based on the literature, four of the genes (*Atf4*, *Hmgn2*, *Vsnl1*, and *Usp29*) were alcohol-related

**Fig. 2:**
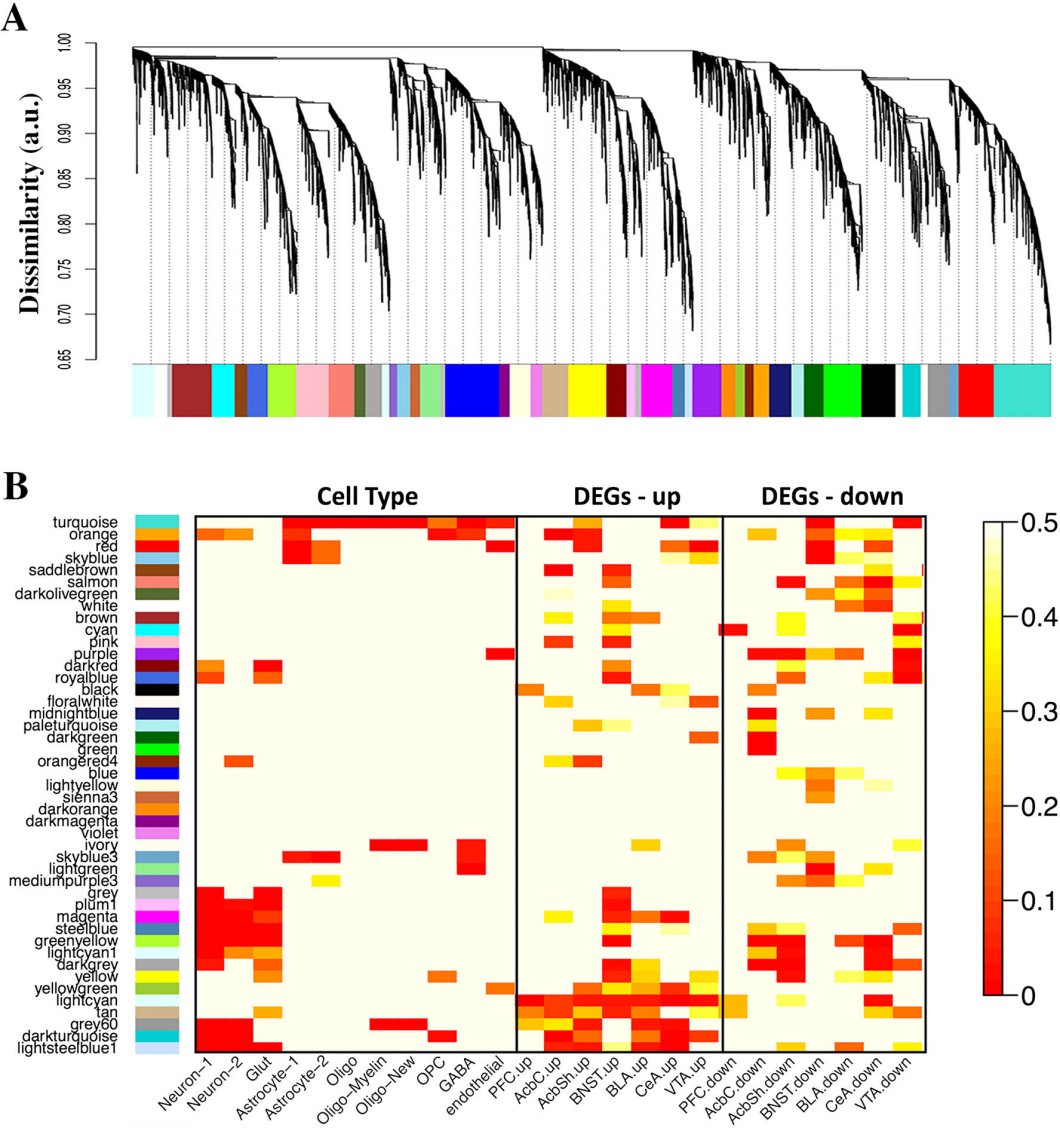
Network analysis of the HDID-1 and HS/Npt transcriptome and functional annotation of gene modules. The dendrogram of the gene network was constructed using all brain regional data from HDID-1 and HS/Npt mice (*N* = 12 mice/genotype/brain region; 12 mice × 2 genotypes × 7 brain regions − 2 outlier samples = 166 samples total) (**a**). The *x*-axis corresponds to genes detected across all regions, and the *y*-axis represents the coexpression distance in arbitrary units (a.u.) between genes, determined by the extent of topological overlap. Dynamic tree cutting identified modules, generally dividing them at significant branch points in the dendrogram. Genes in the 44 modules are color-coded, and those not assigned to a module are labeled gray. Heatmap plots of the false discovery rate (FDR)-corrected hypergeometric *p* values from the over-representation (enrichment) analysis for the differentially expressed genes (DEGs) and cell type-specific genes (**b**). Each row in the heatmap corresponds to one module (labeled by color on the left), and each column corresponds to the category being tested for over-representation. The scale bar on the right represents hypergeometric *p* values used to assess statistical significance of over-representation (red = high statistical significance). *P* values were adjusted using FDR correction. Rows for the DEGs were arranged by hierarchical clustering, and the same row order was maintained for the cell type panel. R was used for analyses and graphical representations

To discern meaningful patterns from the diverse transcriptional response to genetic selection, we used current knowledge databases to perform functional enrichment analysis on the list of 4,121 overall DEGs and the separate DEGs in each brain area (see “Materials and Methods”). The 4,121 DEGs were enriched with neuron-associated terms, such as neuron projection (*q* = 8.12E-30) and synaptic transmission (*q* = 1.97E-10). Many of these DEGs were GABA-A (*Gabrb1*, *Gabrb2*, *Gabrb3*, *Gabrd*, *Gabrg1*, *Gabrg2*) and glutamate receptor subunits (*Gria1*, *Grin1*, *Grik1*, *Grik5*, *Grm5*). Additional DEGs involved in the glutamate/GABA-glutamine cycle included the enzymes producing glutamate, GABA, and their common precursor, glutamine (*Gls*, *Gls2*, *Gad1*, *Glul*), and an excitatory amino acid transporter important for terminating excitatory synaptic transmission (*Slc1a3*). Ingenuity Pathway Analysis (IPA) identified several glutamate- and GABA-related molecular networks, suggesting a shift in balance between excitatory and inhibitory transmission, specifically in the AcbSh (Fig. 1d). The enrichment results are shown in Tables S2 and S3 in Online Resource 2. Additionally, IPA suggested the involvement of dopaminergic systems in selection-responsive gene expression, as levodopa (l-DOPA), the precursor to the catecholamines (dopamine, norepinephrine, and epinephrine), was predicted to be an upstream regulator of the DEGs in every brain region (Fig. 2 in Online Resource 1). The comparison analysis also revealed enrichment of immune-related pathways in most brain regions, such as antigen presentation (*Hla-a*, *Psmb6*) and the complement system (*C1qb*, *C1qc*) (Table S4 in Online Resource 2).

### Identification of Selection-Responsive and Cell Type-Specific Gene Coexpression Networks

A gene network analysis was performed to group genes that share similar expression patterns across the brain regions and genotypes. Expression data were combined and the increased variability in gene expression was used to identify robust correlations and to determine the biological significance of gene networks. The network approach partitioned the diversity of transcriptional responses to genetic selection into several modules of correlated genes (Fig. 2a). This dimensionality reduction shifted the focus from individual genes to gene networks, representing specific cell types and biological functions, and provided a systems-level measure of the effects of genetic selection on behavior. Twenty-five of 44 modules were significantly enriched with the DEGs from at least one brain region (Fig. 2b and Tables S5 and S6 in Online Resource 2), and we refer to them as the *selection-responsive modules*. A functional over-representation analysis was performed for each selection-responsive module (Table 2, Table S7 in Online Resource 2, and Table S8 in Online Resource 3). Each module was over-represented with one or more functional groups or molecular pathways.

**Table 2:** Functional overrepresentation analysis for selection-responsive modules

Upstream regulators			Canonical pathways		
Upstream regulator	No. modules	Module names	Canonical pathway	No. modules	Module names
l-dopa	7	Darkred, Greenyellow, Lightcyan1, Lightsteelblue1, Plum1, Darkgrey, Salmon	Axonal guidance signaling	6	Darkred, Greenyellow, Darkturquoise, Lightcyan1, Magenta, Grey60
WNT3A	5	Darkred, Turquoise, Purple, Grey60, Lightsteelblue1	AMPK signaling	5	Darkred, Royalblue, Darkgrey, Magenta, Purple
Calmodulin	5	Magenta, Darkred, Lightcyan1, Lightsteelblue1, Darkgrey	cAMP-mediated signaling	5	Darkred, Greenyellow, Darkturquoise, Lightcyan1, Plum1
CREB1	5	Magenta, Greenyellow, Lightcyan1, Lightsteelblue1, Darkgrey	CREB signaling in neurons	4	Darkred, Greenyellow, Darkturquoise, Lightcyan1
BDNF	5	Magenta, Darkred, Greenyellow, Grey60, Plum1	Androgen signaling	4	Darkred, Greenyellow, Darkturquoise, Lightcyan1
HDAC4	5	Darkred, Greenyellow, Lightsteelblue1, Plum1, Royalblue	Ceramide signaling	4	Darkred, Royalblue, Darkturquoise, Darkgrey
PPARA	4	Turquoise, Red, Green, Darkturquoise	Gαs signaling	4	Darkred, Royalblue, Darkturquoise, Lightcyan1
Lipopolysaccharide	4	Magenta, Darkred, Turquoise, Red	Dopamine receptor signaling	4	Darkturquoise, Lightcyan1, Darkgrey, Darkgreen
Tretinoin	4	Darkred, Turquoise, Lightsteelblue1, Red	G protein-coupled receptor signaling	4	Darkred, Greenyellow, Darkturquoise, Lightcyan1
Cg	4	Darkred, Turquoise, Purple, Orange			
MYC	4	Magenta, Turquoise, Darkgreen, Green			
CTNNB1	4	Magenta, Darkred, Turquoise, Grey60			
XBP1	4	Turquoise, Darkgreen, Lightgreen, Skyblue			
STAT5A	4	Magenta, Purple, Grey60, Red			
5-Fluorouracil	4	Midnightblue, Darkgreen, Lightgreen, Green			
CD28	4	Purple, Midnightblue, Grey60, Darkgreen			
3-Nitropropionic acid	4	Midnightblue, Grey60, Darkgreen, green			
Ca2+	4	Magenta, Greenyellow, Lightsteelblue1, Plum1			
GATA6	4	Magenta, Purple, Grey60, Green			
KMT2A	4	Magenta, Purple, Greenyellow, Lightsteelblue1			

We identified the functional enrichment of the modules using Ingenuity Pathway Analysis (IPA, see “Materials and Methods”). The reference set for the Fischer’s Exact Test (FET) calculation in IPA was comprised of the transcripts used to construct the coexpression network. We calculated Benjamini-Hochberg’s FDR *q* values to correct for multiple tests, and those with *q* value < 0.05 were considered significant. Each module was over-represented with one or more functional groups or molecular pathways, pointing to underlying biological causes of gene coexpression (*N* = 11-12 mice/genotype)

We used published cell type-specific databases to assign cellular identity to selection-responsive modules. Cell type is the main source of variability in brain gene expression data [11–15]. Thus, modules often represent specific cell populations, which can, in part, be verified by the presence of cell type-specific molecular markers. Twelve of the 25 modules were considered *cell type-specific*, i.e., enriched with genes preferentially expressed in neurons, astrocytes, oligodendrocytes, or brain endothelial cells at a strict false discovery rate (FDR) of < 5% (Fig. 2b and Tables S5 and S6 in Online Resource 2). Established markers of oligodendrocytes (e.g., *Mag*, *Mog*, *Mobp*, *Mbp*, *Ugt8a*, *Cldn11*, and *Trf*), astrocytes (e.g., *Aldh1l1*, *Slc25a18*, *Pla2g7*, *Fgfr3*, *Slc39a12*, *Cyp4f14*, and *Ppp1r3c*), neurons (e.g., *Mef2c*, *Gpr88*, *Myt1l*, *Pcsk2*, *Stmn2*, *Syt1*, and *A130090K04Rik*), and endothelial cells (e.g., *Pecam1*, *Flt1*, and *Vwf*) were present in the cell type-specific modules. The majority of modules were over-represented with biological categories characteristic of the associated cell type, e.g., “synaptic transmission” for neurons or “axon ensheathment” for oligodendrocytes, further validating our systems approach (Table S8 in Online Resource 3).

The selection-responsive modules not associated with a cell type were enriched with terms associated with cell organelles (e.g., nucleus, mitochondrial function, ribosome/protein translation, and primary cilium as well as RNA processing, fatty acid metabolism, immune response, and histone methyltransferase activity). Modules related to housekeeping function had lower variability across brain areas as reflected by their eigengene average expression pattern, whereas neuron-specific modules had higher variability across brain areas, highlighting functional differences among neuronal populations (Tables S5 and S9 in Online Resource 2).

A neuroimmune component was especially prominent, as 12 out of 25 modules had an immune-related function in their top three enriched pathways or biological terms, including Engulfment of Antigen Presenting Cells, IL-1 Signaling, Complement System, IL-17 Signaling, and B Cell Receptor Signaling (Table S5 in Online Resource 2 and S8 in Online Resource 3). Furthermore, lipopolysaccharide (LPS), a component of gram-negative bacteria that activates the innate immune response via activation of toll-like 4 receptors, was predicted by IPA to be an upstream regulator of the turquoise, red, magenta, and darkred selection-responsive modules, suggesting that basal gene expression patterns in HDID-1 brain may mimic an immune response (Table 2 and Table S7 in Online Resource 2). Using our gene expression dataset from C57BL/6J mouse brain after systemic delivery of LPS [16], we found that LPS-regulated genes were enriched in the red, magenta, and darkred modules, confirming the IPA results (Table S10 in Online Resource 2).

### From Gene Networks to Neural Networks: a Systems Approach to Generate Circuit-Level Hypotheses Based on Gene Coexpression

**Fig. 3:**
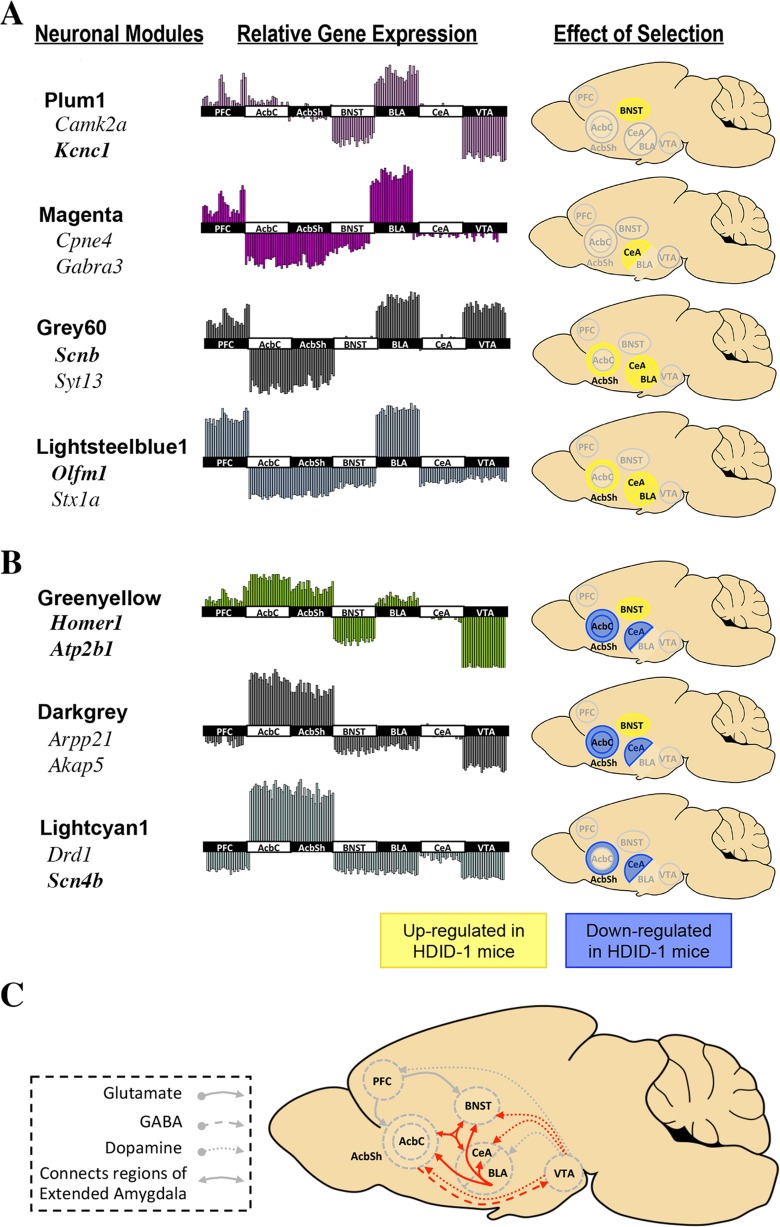
Neuron-specific selection-responsive modules. Shown are the cumulative results for seven modules (networks) highly enriched with neuronal genes. Modules enriched with genes highly expressed in the PFC and BLA and mainly up-regulated in BLA and the extended amygdala (BNST, CeA, AcbSh) (**a**, Group 1). Modules enriched with genes highly expressed in the nucleus accumbens and mainly down-regulated in the AcbSh and CeA (**b**, Group 2). The gene symbols shown under the module name are examples of known neuronal genes that are in the top 20% of the intramodular connectivity values (i.e., hub genes). Genes in bold are differentially expressed between HDID-1 and HS/Npt mice. The plots under “Relative Gene Expression” show the eigengene expression for each module, i.e., average gene expression levels across all genes in a given module. The *y*-axis shows arbitrary expression values, and the *x*-axis shows samples (brain areas are labeled on axis). AcbC, nucleus accumbens core; AcbSh, nucleus accumbens shell; BLA, basolateral amygdala; BNST, bed nucleus of the stria terminalis; CeA, central nucleus of the amygdala; PFC, prefrontal cortex; VTA, ventral tegmental. Direction of regulation in each brain region for each module is shown in color on the schematized brain on the right under “Effect of Selection.” Based on gene coexpression/coregulation results and neurocircuitry literature, we identified neural networks potentially involved in regulation of binge drinking in HDID-1 mice, indicated as red arrows. All of the possible inter-regional connections are not shown (**c**).

A systems approach was used to predict functional neurocircuit changes in the genetically selected HDID-1 mice. Our investigation focused on the eight selection-responsive modules that were highly enriched with neuron-specific genes (*neuronal modules*). Strikingly, all eight modules showed enrichment with DEGs in at least one region of the extended amygdala (seven in CeA, five in AcbSh, and three in BNST), which suggested a critical role for this structure in the regulation of binge drinking (Table S5 in Online Resource 2). Network analysis separated neuronal modules into two general groups with distinct patterns of gene expression and regulation (Fig. 3). Group 1 contained four modules with higher expression in PFC and BLA and consistent up-regulation in at least one of the regions of the extended amygdala and/or BLA (Fig. 3a). Group 2 contained three modules with higher expression in the nucleus accumbens and regulation in the extended amygdala (i.e., down-regulation in the nucleus accumbens and CeA as well as up-regulation in BNST (Fig. 3b). The eighth module (darkturquoise) showed highest expression in the VTA and up-regulation in the nucleus accumbens and CeA (Table S5 in Online Resource 2).

Several criteria were used to generate a circuit-level view of responses to genetic selection for ethanol binge drinking. First, we hypothesized that gene coregulation in two or more brain regions within a neuron-specific module/group suggests the involvement of either direct neural connections between these brain regions or a third region upstream of the coregulated areas. Second, neuronal molecular markers were used to identify specific populations in local or inter-regional neurocircuits. Third, knowledgebases (e.g., IPA) were used to predict “upstream regulators” of selection-responsive, neuron-specific modules (e.g., neurotransmitters, such as dopamine and GABA). Finally, we used published findings to establish inter-regional connectivity among the seven brain regions and to identify neural connections potentially involved in the regulation of drinking in HDID-1 mice based on the first three criteria. Results of these analyses are summarized in Fig. 3c.

Group 1 modules were enriched with genes expressed in glutamatergic projection neurons, e.g., calcium/calmodulin dependent protein kinase II alpha, module plum1 [17] (*Camk2a*) and syntaxin 1A, module lightsteelblue1 [18] (*Stx1a*) (Fig. 3a). These genes also had consistently higher expression in PFC and BLA, brain areas with a large number of glutamatergic projection neurons. This suggested that these neurons are a main source of gene regulation in group 1 modules. Genes within grey60 and lightsteelblue1 modules were up-regulated in BLA, CeA, and AcbSh, which suggested that selection for binge drinking increased glutamatergic signaling from the BLA to the extended amygdala of HDID-1 mice. Additionally, up-regulation of neuropeptide Y, module plum1 (*Npy*) and corticotropin releasing hormone binding protein, module magenta (*Crhbp*) in the extended amygdala suggested the involvement of GABAergic interneurons that may be activated by glutamatergic input [19, 20].

Group 2 modules may, at least in part, represent molecular signaling in medium spiny GABAergic projection neurons (MSNs) in the nucleus accumbens. This is based on high expression values in this region in all three modules and the presence of salient MSN markers, such as the sodium voltage-gated channel beta subunit 4 (*Scn4b*), dopamine- and cAMP-regulated neuronal phosphoprotein, DARPP-32 (*Ppp1r1b*) [21, 22], and the dopamine D1 receptor (*Drd1*) in the lightcyan1 module. Bioinformatics analysis using IPA predicted that both l-DOPA and dopamine were upstream regulators of gene expression in all group 2 modules (Tables 2 and S7 in Online Resource 2), which implicated the dopaminergic system in regulating gene expression in the extended amygdala of HDID-1 mice. Corroborating this finding, all three modules were associated with functional terms related to dopamine function and neuronal plasticity (e.g., dopaminergic synapse, dopamine receptor signaling, long-term potentiation, dendritic spine, and response to amphetamine; Table S7 in Online Resource 2 and S8 in Online Resource 3). Overall, these data suggest that the interplay between glutamatergic and dopaminergic signaling in the extended amygdala (AcbSh in particular) plays a central role in regulating ethanol binge-like drinking in HDID-1 mice. Neural connections shown as red arrows in Fig. 3c help depict the different hypotheses implicating projection neurons between different brain areas in the regulation of ethanol drinking. We tested one of these hypotheses derived from our systems approach as described below.

### HDID-1 Mice Exhibit Altered Neuronal Plasticity to Alcohol Exposure

Based on our analysis and reports from the literature, we hypothesized that HDID-1 and HS/Npt mice differ in functional properties of MSNs in the nucleus accumbens shell projecting to the VTA. For example, the selection-responsive, neuron-specific lightcyan1 module (Fig. 3b) contained MSN markers (*Drd1* and *Ppp1r1b*) and showed overall down-regulation of gene expression in the AcbSh. A large portion of Drd1-containing, *but not* Drd2-containing AcbSh MSNs, send projections to the VTA as part of the “direct” striato-tegmental pathway implicated in the regulation of goal-directed behaviors [23, 24]. Dopamine regulation, putatively originating from VTA neurons, was also predicted based on gene expression in the lightcyan1 module (Table S7 in Online Resource 2). In addition, the lightcyan1 module represented the “hub module,” i.e., genes in this module had the highest number of connections with genes in other modules (Table S11 in Online Resource 2). The other two modules in group 2 were in the top four hub modules (Table S11 in Online Resource 2). Like hub genes, hub modules may be particularly essential to the function of the network. These findings suggest the involvement of Drd1-containing AcbSh MSNs projecting to the VTA in the regulation of binge drinking in HDID-1 mice. Down-regulation of *Scn4b*, which is known to modulate accumbal long-term depression (LTD) [25], provides additional support for our hypothesis and eludes to differences in synaptic plasticity between HDID-1 and control mice. Our group has previously shown that chronic intermittent ethanol (CIE) vapor exposure attenuates LTD in AcbSh Drd1-containing MSNs in genetically modified mice, suggesting that this ethanol-mediated neuronal adaptability may contribute to the neuroadaptations underlying the development of ethanol dependence [26].

**Fig. 4:**
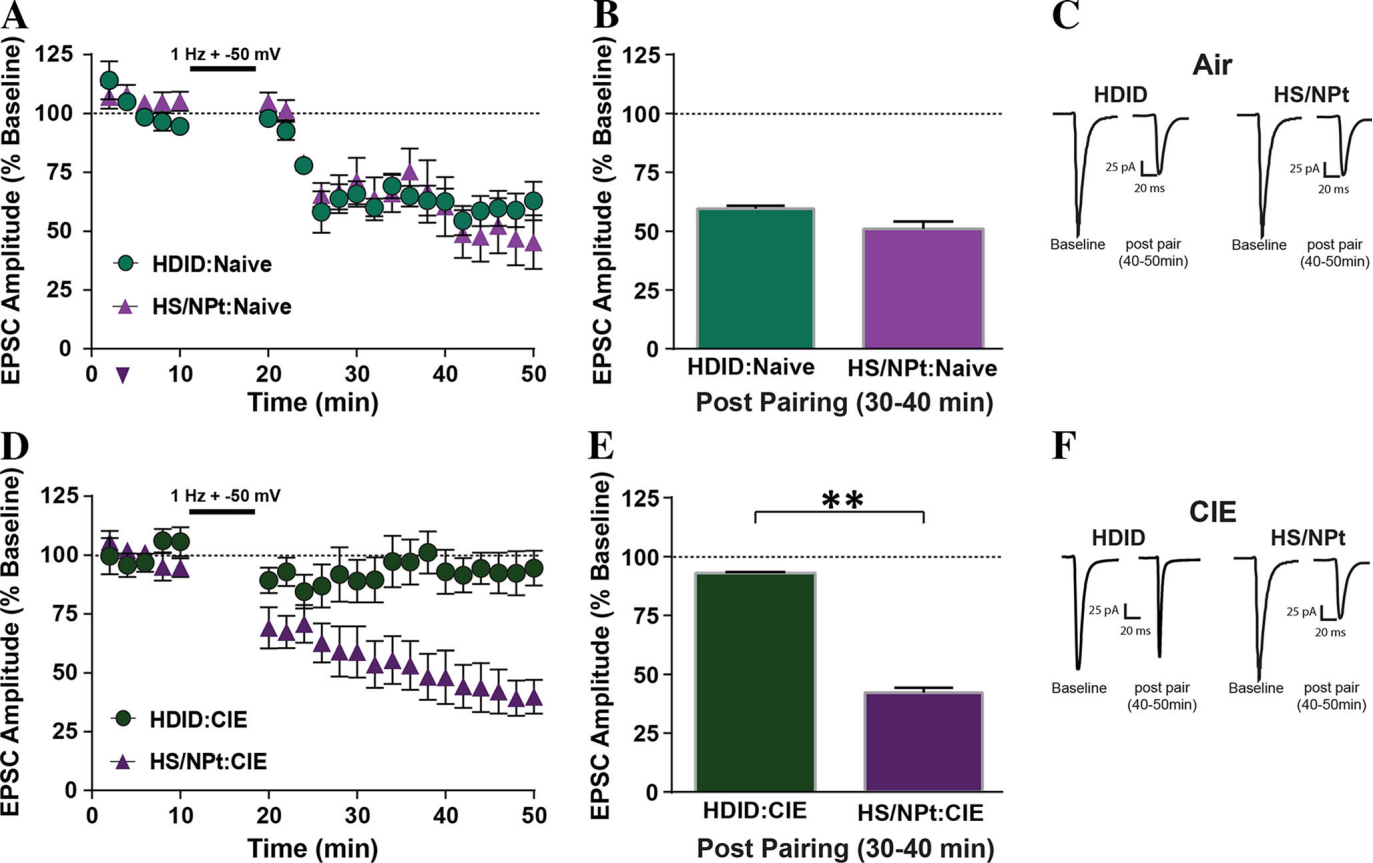
Accumbal plasticity in D1+ medium spiny neurons (MSNs) before and after the chronic intermittent ethanol (CIE) procedure in HDID-1 and HS/Npt mice. The pairing protocol (1 Hz stimulation paired with depolarization to − 50 mV) induced LTD in Drd1-MSNs in air-treated HDID-1 and HS/Npt mice (**a**). Bar graphs representing the percentage change ± SEM for average EPSC amplitude post-pairing (40–50 min) as a percentage of baseline (0–10 min) in Drd1-MSNs for HDID-1 (61.89 ± 1.6, *N* = 7) and HS/Npt (48.61 ± 2.52, N = 7) mice (**b**). Sample EPSCs during baseline and post-pairing (40–50 min) in Drd1-MSNs of HDID-1 and HS/Npt mice (**c**). Twenty-four hours after 4 days of CIE (16 h/day), the pairing protocol produced occlusion of LTD in Drd1-MSNs of HDID-1 mice but not in HS/Npt mice (**d**). Bar graphs representing the percentage change ± SEM for average EPSC amplitude post-pairing (40–50 min) as a percentage of baseline (0–10 min) in Drd1-MSNs of HDID-1 (92.11 ± 0.84, *N* = 6) and HS/Npt (41.75 ± 2.02, N = 6) mice 24 h after CIE (**e**). Sample EPSCs during baseline and post-pairing (40–50 min) of Drd1-MSNs of CIE-treated HDID-1 and HS/Npt mice (**f**). Scale bars represent 25 pA (vertical) and 20 ms (horizontal). **p < 0.01 versus baseline

To test this circuit-level hypothesis, we conducted whole-cell, patch clamp recordings in AcbSh MSNs (labeled with a retrograde tracer injected into the VTA) of ethanol-naïve and ethanol-exposed mice. No differences in LTD induction were found between ethanol-naïve HDID-1 and HS/Npt mice (Fig. 4a–c), whereas 4 days of CIE vapor exposure produced a loss of LTD expression in MSNs isolated from HDID-1 but not HS/Npt mice (Fig. 4d–f), suggesting ethanol-induced differences in synaptic plasticity between the genotypes.

### HDID-1 Mice Share Coexpression Patterns with Human Alcoholics

**Fig. 5:**
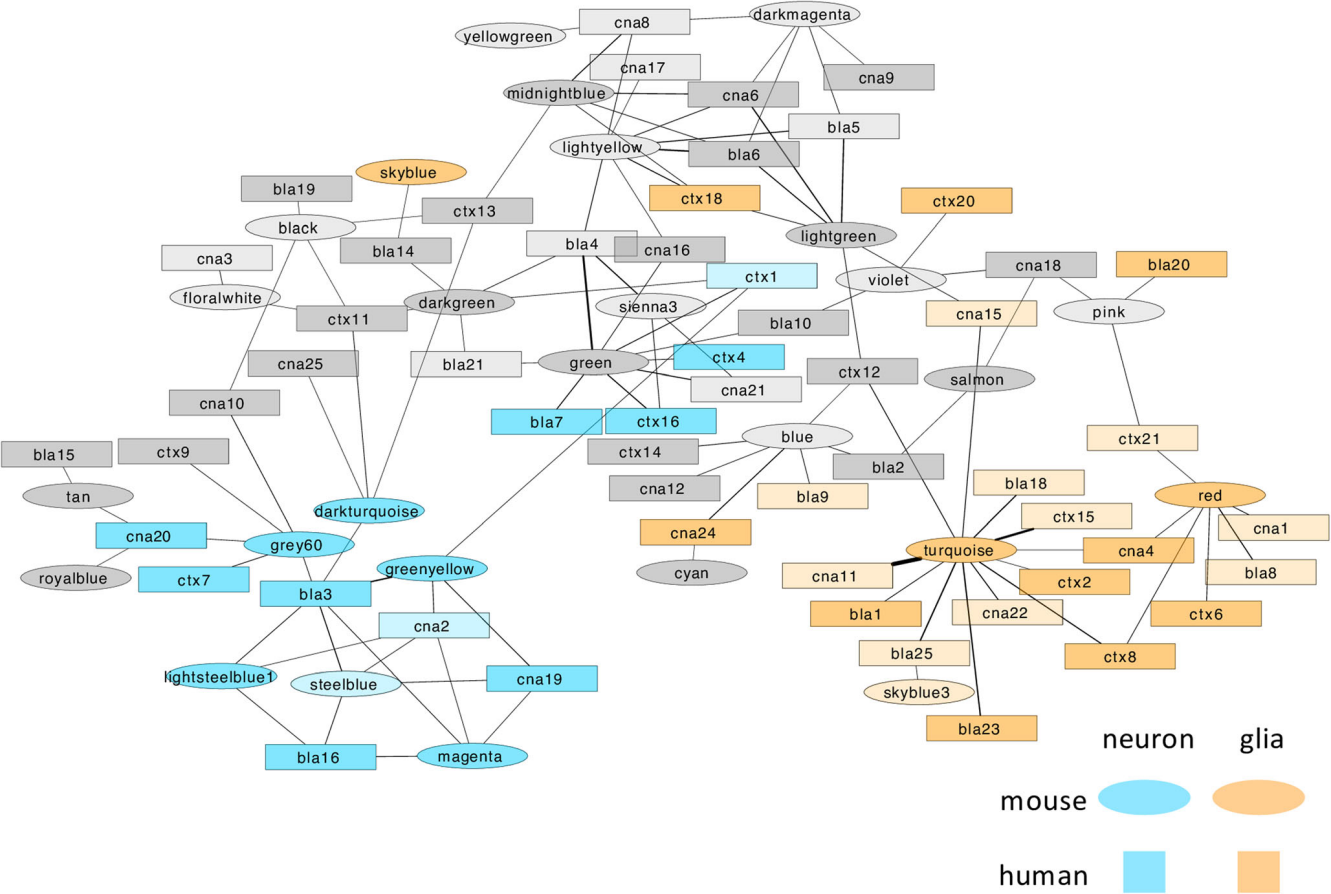
Comparison of HDID-1 and human alcoholic gene networks. A visualization of the interspecies network comparison of human and mouse created with Cytoscape 3.2.1. The nodes are modules from the mouse (circles) or human (squares) networks. The edges are the –log10 values of the hypergeometric *p* values of the number of overlapping probes between two nodes (modules). Edge thickness is proportional to the statistical significance of the overlap between the nodes. Only highly overlapping modules are shown (hypergeometric *p* < 0.001). There is a strong separation of nodes based on their enrichment with cell type-specific genes (see “Materials and Methods”), with neuronal (blue) and glial (orange) modules clustering together, independent of species. Most of the highly overlapping modules in these clusters are alcohol-related (i.e., enriched with genes differentially expressed between human alcoholics and controls or HDID-1 mice and controls), denoted by the intensity of the color (more intense color indicates alcohol-related).

Integration of large-scale “omics” data across species and animal models is critical for our understanding of normal behavior and brain disorders. To identify common molecular networks associated with high alcohol consumption, we conducted an interspecies comparison of gene coexpression patterns between HDID-1 mice and human alcoholics [10]. Similar to previous studies [10, 11, 13, 27–32], there was considerable conservation of gene coexpression between mice and humans (i.e., the modules were largely comprised of the same genes) (Figs. 5 and S3 in Online Resource 1). Highly overlapping, or conserved, networks were identified between species (29/44 modules in the mouse network and 55/72 modules in the human network; hypergeometric *p* value < 0.001). Importantly, 17/25 mouse selection-responsive modules overlapped with 29/46 human alcohol-related modules (Fig. 5, hypergeometric *p* value < 0.001), suggesting that the HDID-1 mouse model partially reproduced the transcriptomic dysregulation observed in human alcoholics.

There was significant preservation of glial and neuronal modules between mouse and human networks, revealing separate neuronal and glial sub-networks (Fig. 5). Modules related to cellular organelles (such as nuclear, mitochondrial, and ribosomal functions) were also conserved, consistent with previous studies [30]. To highlight cell types and biological functions relevant to AUD in humans, we focused on mouse modules that were (1) selection-responsive, (2) conserved between species, (3) cell type-specific, and (4) connected to alcohol-related, cell type-specific human modules. Five neuronal and two glial selection-responsive modules met these criteria (Fig. 5).

As expected, mouse modules that were enriched with glial markers overlapped with astrocyte, microglial, and oligodendrocyte modules in the human network. IPA of the overlapping genes between the human alcohol-related modules and mouse selection-responsive modules in the glial sub-network showed over-representation with many biological categories indicative of glial functions (e.g., NF-kB Signaling, Th1 and Th2 Pathways, LPS-IL-1 Inhibition of Retinoic X Receptor Function, Granulocyte Adhesion, and Diapedesis) as well as typical endothelial functions (e.g., eNOS Signaling). Other enriched pathways included Estrogen Biosynthesis, Fatty Acid β-oxidation, Dopamine Degradation, and p53 Signaling (see Table S12 in Online Resource 2).

**Fig. 6:**
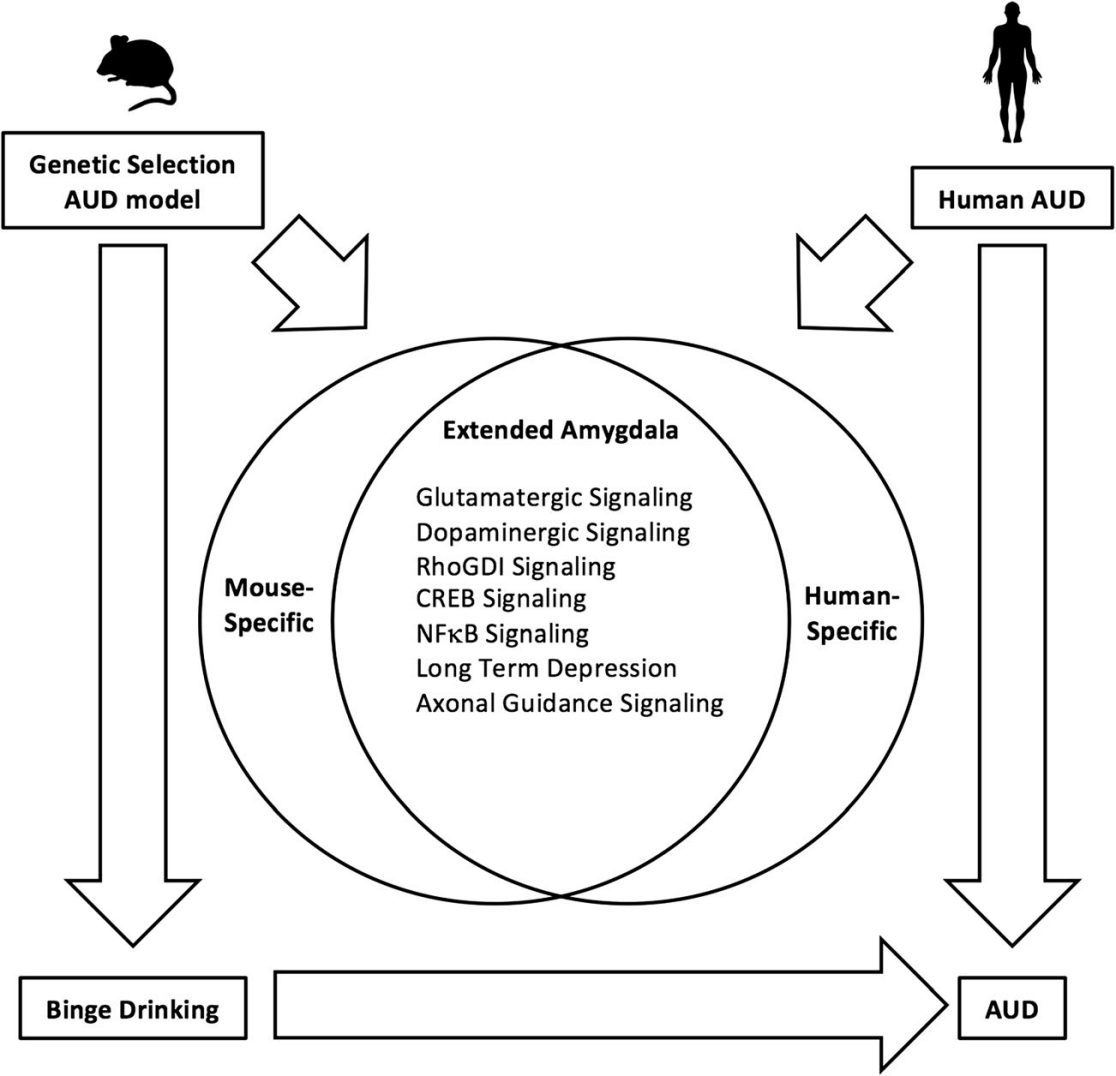
A hypothetical diagram based on comparative transcriptome analysis of a mouse model of binge drinking and people diagnosed with alcohol dependence. Our systems-level analyses revealed gene expression patterns that are “conserved” between alcoholics and HDID-1 mice, implicating several molecular functions in specific cell types in driving AUD risk

The overlapping human and mouse genes in the neuronal sub-network were predictably enriched with molecular pathways involved in synaptic plasticity, including glutamate receptor signaling, axonal guidance signaling, long-term depression, CREB signaling in neurons, and RhoGDI signaling (e.g., *Gria1*, *Gria2*, *Homer1*, *Homer2*, *Itpr*, *Grik5*, *Arhgdig*, *Arhgef11*, *Arhgap1*; Table S12 in Online Resource 2). Additionally, the overlapping genes in the neuronal sub-network were predicted to be downstream of l-DOPA, and 13/38 of these genes were differentially expressed in both human alcoholic brain and HDID-1 mouse brain (*Aspscr1*, *Ctnnbip1*, *Egfl7*, *Gabrg2*, *Hpca*, *Lmtk2*, *Nuak1*, *Palm*, *Rasgrp1*, *Slc6a7*, *Sncb*, *Tiam2*, and *Tpd52*; Table S12 in Online Resource 2). Although the accumbens was not part of the human study, the alcohol-related CeA modules (part of the extended amygdala) were integrated in the conserved network. Overall, this analysis suggested that patterns of excessive drinking in the genetic mouse model and human alcoholics have some common underlying mechanisms and that glutamatergic and dopaminergic signaling within the extended amygdala are points of interspecies convergence (Fig. 6).

## Discussion

Binge drinking is an important risk factor in the development of AUD, and HDID-1 mice effectively model this pattern of drinking. In fact, HDID-1 mice fulfill many of the criteria proposed for a model of excessive alcohol consumption [33] (reviewed in [34]). Here we characterized gene expression, a molecular phenotype, across seven brain regions in ethanol-naïve HDID-1 mice. Our results confirm and extend findings from a previous gene expression study in the ventral striatum from ethanol-naïve HDID-1 mice [10]. However, unlike this study, we used a neurocircuit-specific approach to generate and test circuit-level hypotheses. There was a clear convergence of molecular phenotypes in HDID-1 mice and human alcoholics, and the identified genes provide potential targets for modifying binge-like alcohol consumption. The mice were ethanol-naïve, suggesting that the convergent molecular functions between the mouse and human networks are important for driving AUD risk, rather than a consequence of chronic alcohol use, and represent potential therapeutic targets for AUD. Selective breeding for attaining high BALs fixed the genetic alleles related to the DID phenotype in HDID-1 mice. However, due to genetic drift some alleles that are unrelated to binge drinking could also be driven to fixation, potentially affecting gene expression. This possibility could be addressed by testing the second replicate line, HDID-2, which may provide further validation for convergent molecular functions between species.

A novel component of our systems genomic approach was to use correlated patterns of gene expression in multiple brain regions to define neural networks that potentially regulate alcohol drinking in HDID-1 mice. This approach was based on the concept that brain regions with correlated patterns of gene expression and regulation also have synchronized patterns of neuronal activity. A recent test of this hypothesis showed that functional brain networks, represented by resting-state fMRI, are recapitulated using measures of correlated gene expression in a *postmortem* brain tissue dataset [35]. Our analysis provided additional support for this concept, as we were able to formulate circuit-level hypotheses based on gene expression and validate one of the predicted neural connections experimentally. There are different methods to generate neurocircuit hypotheses based on gene expression, including direct gene correlations across brain regions as well as constructing and overlapping gene networks from individual brain areas; future studies are needed to determine the predictive validity of each approach.

Our initial hypothesis focused on the reciprocal connections among regions of the extended amygdala and the VTA given that optogenetic and chemogenetic studies showed activation or inhibition of some of these neural pathways altered ethanol consumption [19, 36, 37]. We found that accumbal MSNs projecting to the VTA showed differential synaptic plasticity in HDID-1 and control mice after dependence and withdrawal from ethanol. We expected to find genotypic differences in VTA-projecting Drd1-containing AcbSh MSNs in *both* ethanol-naïve and ethanol-exposed mice. The lack of changes in synaptic plasticity in naïve animals and the divergent responses after ethanol suggest that genotype differences in expression translate into functional differences *only* after “priming” with ethanol. The “priming” hypothesis is supported by a study showing that activation of Drd1-expressing MSNs in the nucleus accumbens altered basal locomotor activity and conditioned place preference only after “priming” with cocaine [38]. We hypothesize that ethanol differentially alters the functional activity of accumbal Drd1-expressing MSNs in HDID-1 and HS/Npt mice, and the transcriptional effects of genetic selection become evident after repeated drug exposure. CIE exposure in HDID-1 mice increases binge-like drinking [39], and ethanol-induced changes in MSN LTD may contribute to this behavior.

Altered neuronal synaptic plasticity in response to environmental challenges, such as ethanol, is referred to as metaplasticity, which may be defined as the “plasticity of synaptic plasticity” [40]. Metaplasticity allows synapses to integrate synaptic signals across time. In terms of long-term potentiation (LTP) or LTD, metaplasticity may involve changes in induction thresholds as a means to regulate synaptic signaling strength. Unlike control mice, HDID-1 mice exhibited a loss of LTD after ethanol exposure. Alterations in basal metaplastic responses of AcbSh MSNs in HDID-1 mice may confer sensitivity to ethanol-induced plasticity. This is consistent with work showing that ethanol decreased LTD in C57BL/6J mice, a line exhibiting high preference for ethanol [26], and this was associated with increased ethanol intake during withdrawal. Altered metaplasticity in HDID-1 mice may partially underlie dependence-induced increases in binge-like drinking or may increase ethanol withdrawal symptoms and promote drinking relapse.

Glutamate signaling is involved in metaplasticity, and the differential expression of mGluR1 and Homer2 in HDID-1 versus HS/Npt mice could thus contribute to genotype differences in metaplasticity. HDID-1 mice express higher basal protein levels of mGluR1 and Homer2 in the AcbSh [41], and the activity of mGluR1 and Homer2 within this region is critical for binge-like ethanol consumption [42]. Our study did not reveal basal differences in mRNA levels of these proteins; however, *Homer2*, *mGlur1*, *Homer1*, and *PKCε* (a molecule downstream of the mGluR/Homer pathway) were localized in the neuron sub-network that was conserved between HDID-1 mice and human alcoholics. Moreover, *PKCε* and *Homer1* are DEGs in HDID-1 mice and alcoholics. The ratio of p(Ser729)-PKCε to total PKCε at baseline (but not the total PKCε) was higher in HDID-1 compared with HS/Npt mouse CeA. Evidence thus suggests that the mGluR1/Homer/PKCε pathway in the extended amygdala of HDID-1 mice likely contributes to an ethanol-sensitive metaplastic state. Down-regulation of *Scn4b* in the AcbSh of HDID-1 mice may also be a contributing factor. For example, *Scn4b* selectively regulated LTD and removal or down-regulation of this subunit was associated with decreased LTD [25]. Although we did not measure gene expression after ethanol exposure, based on our results showing *Scn4b* down-regulation in ethanol-naïve mice and its role in LTD, ethanol-induced changes in LTD would also be expected.

The conserved patterns of gene expression between HDID mice and human alcoholics revealed a clear separation of neuronal and glial signaling and highlighted three broad functional categories: glutamatergic neurotransmission, genes potentially regulated by l-DOPA/dopamine signaling, and immune-related processes, all of which are implicated in ethanol-related behaviors. Our results suggested a role for GABAergic MSNs in the extended amygdala and glutamatergic neurons in the BLA in the regulation of drinking in HDID-1 mice. There is ample evidence that glutamatergic signaling in the nucleus accumbens is important for ethanol consumption [43, 44]. Changes in the GABA/glutamate-glutamine cycle genes were strongest in the AcbSh and suggest genetic selection for high drinking might augment excitatory neurotransmission in this region. Our systems approach also suggested that transcriptional activation in the BLA may increase glutamatergic signaling in the extended amygdala of HDID-1 mice. Hypothetically, neuroadaptations in HDID-1 mice may confer the ability to consume high amounts of ethanol without experiencing negative effects. This hypothesis is supported by evidence of blunted responses to ethanol’s aversive properties in HDID-1 mice [45]. Aversive stimuli trigger increased glutamatergic signaling from the extended amygdala (specifically the BNST) to VTA [46], and the increased glutamatergic signaling in HDID-1 mice might render them less sensitive to aversive stimuli like ethanol [47]. It is important for future studies to test the effects of genetic or pharmacological manipulation of the glutamatergic signaling genes implicated here (e.g., *Gria1*, *Grm5*, *Grik5*, or *Homer1*) on ethanol-induced aversive behaviors in HDID-1 mice.

Our results also support a neuroimmune mechanism in ethanol-related behavior, as has been observed in many species, including humans, rodents, and flies (for review, see [48, 49]). Dysregulation of neuroimmune genes was found in both HDID-1 mice and alcoholics, and glial, neuronal, and non-cell type-associated modules were enriched with neuroimmune pathways. Genetic selection for DID perturbed many of the same genes that were affected by LPS (as predicted by IPA and enrichment with published datasets of brain gene expression changes 1 week after systemic LPS delivery), which could contribute to dopaminergic changes and a high drinking phenotype. These findings do not necessarily imply that HDID-1 mice have a more active immune system than control mice, as there is not a direct relationship between peripheral and central immune responses. Changes in brain gene expression 1 week after LPS most likely represent downstream effects rather than an acute immune response. We, however, did not sample peripheral tissue or measure serum cytokine levels and cannot exclude the possibility of increased immune activity in HDID-1 mice.

We note that microarray technology is prone to “misreads” due to genetic polymorphisms, such as SNPs, making it possible that some DEGs were due to genotype variations in microarray performance rather than expression levels of mRNA [50, 51]. Therefore, the results for individual probes should be interpreted with caution, especially for those probes that map to areas of the genome that contain known SNPs (see Table S1). This inherent weakness of microarrays could be addressed by validating individual genes using a different platform, including RNA sequencing and quantitative PCR. Our main findings, however, are based on gene networks and the probability of many probes in a given network being affected by genetic polymorphisms is low. We want to highlight the importance of taking a multifaceted approach that incorporates multiple levels of evidence to improve the biological insights compared to single gene approaches. Another potential concern is that the mice used in the gene expression and electrophysiological experiments were from different selection generations and were maintained in different facilities. However, the gene expression data from selection generation S16 predicted circuit-level function in mice from S33, suggesting that the transcriptional signature was still prevalent after 17 additional generations of selection.

In summary, our results support the hypothesis that genetic differences in HDID-1 and HS/Npt mice resemble those between alcoholics and non-alcoholics, particularly in genes related to neuroimmune, dopaminergic, and glutamatergic signaling. We also demonstrated functional differences in neuronal plasticity between HDID-1 and HS/Npt mice following ethanol exposure, supporting the idea that ethanol-induced neural adaptations within the nucleus accumbens are critical for the expression of ethanol-related behaviors [52]. Our systems approach demonstrated how the HDID-1 model can be used to probe mechanisms of binge drinking and how molecular profiling may be extended to the circuit level to identify potential targets for treating AUD.

## Materials and Methods

### Animals

HS/Npt mice are the non-selected and genetically diverse population from which the HDID-1 mice were selected as previously described [6, 53]. Adult (euthanized at age 8–12 and 10 weeks for the gene expression and electrophysiological studies, respectively), experimentally naïve, male HDID-1 (Selected Generation S16 and S33 for the gene expression and electrophysiological studies, respectively) and HS/Npt (Generation G68 and G84 for the gene expression and electrophysiological studies, respectively) mice were bred and housed in the Veterinary Medical Unit at the Veterans Affairs Medical Center (Portland, OR, USA) (*N* = 12 and 18 mice/genotype for the gene expression and electrophysiological studies, respectively). Mice used for functional experiments were shipped from Oregon Health & Science University and acclimated at the College of Pharmacy at The University of Texas at Austin (Austin, TX, USA) for approximately 4 weeks before beginning the ethanol vapor experiments. Mice received ad libitum access to food (Purina 5001 and RMH1800 chow for the gene expression and electrophysiological studies, respectively; LabDiet, St. Louis, MO, USA) and water. Mice were kept on a reverse 12 h/12 h light/dark cycle with lights on at 2130 h for all experiments. All procedures were approved by the local Institutional Animal Care and Use Committee and conducted in accordance with the NIH Guidelines for the Care and Use of Laboratory Animals.

### Tissue Preparation for Gene Expression Analysis

Mice were decapitated and their brains were quickly removed, flash frozen in liquid nitrogen, and stored at − 80 °C then shipped on dry ice to The University of Texas at Austin. Twenty-micron coronal sections were prepared using a Microm HM550 cryostat (Thermo Fisher Scientific, Walldorf, Germany). Serial sections were collected and mounted on UV-treated nuclease-free PEN membrane slides (Zeiss, Bernried, Germany). Slides were kept inside the cryochamber during the procedure and stored at − 80 °C until ready to use.

### Staining Procedures

Sections were quickly transferred from − 80 °C to pre-cooled (4 °C) 95% ethanol and incubated for 1 min, followed by rehydration in pre-cooled (4 °C) 70% ethanol for 20 s. The slides were stained in 1% cresyl violet acetate (Sigma, St. Louis, MO, USA) in absolute ethanol for 40 s and dehydrated for 5 s each in 70 and 95% ethanol and then for 15 s in 100% ethanol at room temperature. After air-drying for 2 min, slides were stored in a slide box with desiccant on ice and immediately used for microdissection.

### Laser Capture Microdissection

Seven brain regions (PFC, AcbC, AcbSh, BNST, BLA, CeA, and VTA) were dissected in accordance with coordinates from the mouse brain atlas (Franklin and Paxinos, 2007) using the PALM MicroBeam system (Carl Zeiss MicroImaging, Bernried, Germany): PFC (bregma = 1.6–2.1 mm, lateral = 0.1–0.4 mm, ventral = 1.5–3.5 mm); AcbC (bregma = 1.0–1.6 mm, lateral = 0.7–1.5 mm, ventral = 4.0–4.7 mm); AcbSh (bregma = 1.0–1.6 mm, lateral = 0.5–1.5 mm, ventral = 4.2–5.2 mm); BNST (bregma = 0.0–0.3 mm, lateral = 0.3–1.1 mm, ventral: 3.8–4.7 mm); BLA (bregma = − 1.8 to − 0.9 mm, lateral = 2.5–3.0 mm, ventral = 4.5–5.0 mm); CeA (bregma = − 1.7 to − 0.8 mm, lateral = 2.0–2.5 mm, ventral = 4.3–4.8 mm); VTA (bregma = − 3.8 to − 3.0 mm, lateral = 0.1–0.9 mm, ventral = 4.0–4.8 mm). Sections containing these brain regions were cut under ×10 magnification and manually transferred using ultrafine forceps into non-stick, nuclease-free 1.5-ml tubes on ice. Several sections were pooled.

### RNA Extraction

Lysis solution from RNAqueous-Micro Kit (Ambion, Austin, TX, USA) was added to the microdissected samples, and samples were incubated at 42 °C for 30 min and stored at − 80 °C until processed. RNA was isolated following the manufacturer’s instructions for laser capture microdissection (LCM) samples and quantified using the Quant-iT RiboGreen RNA Assay Kit (Invitrogen, Carlsbad, CA, USA), and then quality was assessed using the Agilent RNA 6000 Pico Kit (Agilent Technologies, Santa Clara, CA, USA). The average RNA integrity number was 6.77 ± 0.58, indicating that the samples were of good quality. Samples from both genotypes were processed in parallel to avoid potential batch effects.

### RNA Amplification and Hybridization

Total RNA was amplified using the Illumina TotalPrep-96 RNA Amplification Kit (Ambion, Austin, TX, USA). The cRNA was quantified using a NanoDrop ND-1000 spectrophotometer (NanoDrop Technologies, Wilmington, DE, USA) and analyzed using the Agilent RNA 6000 Nano Kit (Agilent Technologies, Santa Clara, CA, USA). Input of 30 ng of RNA yielded at least 4 μg of amplified biotinylated cRNA, with most samples distributing between 800 and 1500 nt, indicating the expected yield and size. We sent 1.5 μg cRNA to The Microarray Resource at Yale University where it was hybridized to Illumina Mouse WG-6 v2.0 Expression BeadChips (Illumina, San Diego, CA, USA). Samples from different genotypes and brain regions were counterbalanced on each chip to avoid potential batch effects. All chips were processed simultaneously.

### Data Preprocessing

The data are publicly available on Gene Expression Omnibus under the accession number GSE9331. We preprocessed the data using the Bioconductor lumi package version 2.20.2 in the R programming environment [54]. A total of 168 samples across seven brain regions were used for data analysis. Variance-stabilizing transformation and quantile normalization were applied [55]. Outlier detection was performed within each brain region, and samples were considered to be outliers when their distances to the sample average were larger than the threshold (2*median distances to the center). Two control samples (from AcbC and BNST) were removed. Variance-stabilizing transformation and quantile normalization were then reapplied to the raw data of the remaining 166 samples. For each brain region, only those genes that were significantly expressed (detection threshold *p* < 0.05) in at least 75% of samples were selected. Grubb’s test (*p* < 0.05) was performed within groups to remove outliers for each gene.

### Differential Expression Analysis

Differential expression analysis was conducted for each brain region by empirical Bayes- moderated *t* statistics using the Bioconductor limma package version 3.24.15 in R [56]. DEGs (*p* < 0.05) in HDID-1 and HS/Npt mice were identified. The number of DEGs in each brain region was compared to chance using a chi-square test to estimate effects of genetic selection on global gene expression. We then compared our DEGs with those of a previous study in ventral striatum of ethanol-naïve, male HDID and HS/Npt mice [10]. We identified Illumina probes that map to areas of the genome containing known single nucleotide polymorphisms (SNPs) using the Bioconductor illuminaMousev2.db package version 1.26.0 in R.

Enriched molecular pathways of the DEGs were identified using two web-based software applications (knowledgebases): IPA (Ingenuity Systems, www.ingenuity.com) and ToppFun (https://toppgene.cchmc.org/enrichment.jsp) [57]. We performed a core analysis for each brain region in IPA using default settings, except that expressed transcripts were used as the background population for the right-tailed Fischer exact test (FET) calculations. Terms with FET *p* < 0.05 were considered significantly enriched within the dataset. For the ToppFun analysis, terms with Benjamini-Hochberg’s FDR *q* < 0.05 were considered significant.

### Gene Network Analysis

We used the WGCNA R package version 1.48 developed by Langfelder and Horvath to perform weighted gene coexpression network analysis on all samples [58] (https://labs.genetics.ucla.edu/horvath/CoexpressionNetwork/Rpackages/WGCNA/). Transcripts detected in all seven brain regions were included in the analysis. The Pearson correlation coefficient between all pairs of probes across all samples was calculated, and a signed gene coexpression similarity matrix between genes was generated: *S*_*ij*_ *=* (1 *+ cor*(*x*_*i*_,*x*_*j*_)) / 2. Then, an adjacency matrix, *a*_*ij*_ *= S*_*ij*_^*β*^, was used to assess gene connections. Power (*β*) was chosen so that the resulting network exhibited approximate scale-free topology (*β* = 12). Next, the topological overlap measure (TOM) was used to calculate the relative interconnectedness of a gene pair. Average linkage hierarchical clustering was applied to produce a dendrogram based on the topological overlap dissimilarity (1 − TOM). Branches of the tree were cut using a dynamic tree cut algorithm to detect modules (deepSplit = *T*, minimum module size = 80, cut height = 0.995). The terms “modules,” “clusters,” and “gene networks” are used interchangeably in the manuscript and refer to groups of genes with highly correlated (positively or negatively) expression levels across samples. A hub gene is a highly interconnected gene within a module. It can be determined by calculating the intramodular connectivity *K*_in_ for each gene by summing the adjacencies of that gene with other genes in that particular module. It measures how correlated a gene is with the others in that module. For each module, we identified hub genes (top 20%).

To determine the network “hub modules,” we labeled each node (i.e., gene) in the network with its module color assignment and calculated the number of connections each module had with a node in a different module normalized by module size. We performed this calculation for all connections and also for the connections in the top quartile.

### Identification of Gene Modules Related to HDID-1 Selection

To identify gene coexpression modules that may drive excessive ethanol consumption in HDID-1 mice (selection-responsive modules), we performed an over-representation analysis of DEGs for each module. Hypergeometric testing was used to assess the significance of module enrichment with up-regulated and down-regulated DEGs from each brain region. Benjamini-Hochberg’s FDR *q* values were calculated to correct for multiple tests (*q* < 0.05 were considered significant).

We also used the module eigengene to identify modules that are related to brain region, i.e., those containing genes enriched or depleted in specific brain regions. One-way ANOVAs of the module eigengene were performed for each module to assess variation between brain regions. A graphic representation of a module eigengene reflects relative gene expression among brain regions (e.g., see Fig. 3).

### Functional Enrichment Analysis

Several complementary approaches were used to characterize the selection-responsive modules. First, we identified modules enriched with established cell type-specific markers using the hypergeometric test. Expressions of genes with ≥fourfold enrichment in astrocytes, oligodendrocytes, and neurons [59] were considered to be cell type markers. We used the top 500 DEGs in the following cell types (relative to the other cell types in the study) as additional cell type markers (neurons, microglia, astrocytes, and oligodendrocytes at different maturation states as well as endothelial cells) using a minimum Fragments Per Kilobase of transcript per Million mapped reads (FPKM) threshold of 20 (http://web.stanford.edu/group/barres_lab/brain_rnaseq.html) [60]. Gene datasets preferentially expressing glutamatergic and GABAergic neuronal subpopulations were used to further characterize the neuronal modules [61]. Second, we identified the functional enrichment of the modules using IPA and ToppFun as described above. The reference set for the FET calculation in IPA was comprised of the transcripts used to construct the coexpression network. Benjamini-Hochberg’s FDR *q* values were calculated to correct for multiple tests, and those with *q* value < 0.05 were considered significant [62].

We used two publicly available databases to link DEGs and selection-responsive modules with alcohol consumption: (1) INIA Texas Gene Expression Database (IT-GED, http://inia.icmb.utexas.edu) containing curated lists of significantly regulated genes from microarray studies focusing on models of excessive alcohol consumption and (2) Ethanol-Related Gene Resource (ERGR; https://bioinfo.uth.edu/ERGR/) containing more than 30 datasets, including linkage, association, and microarray gene expression from the literature and 21 mouse QTLs from public databases [63].

### Mouse-to-Human Network Comparisons

We compared the HDID-1-HS/Npt mouse gene networks with those constructed from human alcoholics/controls [10]. The illuminaHumanv3.db R package version 1.26.0 was used to update the annotations to the human genome release, hg19, from the UCSC genome browser. We converted mouse gene symbols to their corresponding human orthologues by cross-species mapping using BioMart (http://www.ensembl.org/biomart/martview). The numbers of genes shared among human BLA, central nucleus of the amygdala (CNA), and superior frontal cortex (CTX) and the mouse network are 6290, 6330, and 6436, respectively. The hypergeometric distribution was used to assess the significance of internetwork module overlap. We used Cytoscape to visualize the network comparisons with meta-network graphics and showed highly significant connections (hypergeometric *p* < 0.001) [64].

### Labeling of Drd1-MSNs

A subset of HDID-1 and HS/Npt mice (5 weeks old) used for behavioral and electrophysiological experiments underwent stereotaxic injection of the retrograde tracer, Alexa Fluor 555 labeled recombinant cholera toxin subunit B (CTB; Molecular Probes) into the VTA. Anesthesia was induced at 3% and maintained at 1.5% isoflurane (*w*/*v*) (Baxter AG) during surgery. Animals were placed in a stereotaxic frame (Kopf) and bilateral craniotomies were performed using stereotaxic coordinates adapted from the Paxinos & Watson mouse brain atlas (for VTA: anterior–posterior = − 3.1; medial–lateral = ± 0.38; dorsal– ventral = − 4.8). Injections of CTB (0.5 μl per injection site) were made using graduated pipettes (Drummond Scientific), broken back to a tip diameter of 10–15 μm, at an infusion rate of ≈ 0.05 μl/min. CTB injections were performed a minimum of 4 weeks before ethanol exposure.

### Chronic Intermittent Ethanol Exposure

Mice were exposed to ethanol vapor using the CIE model [26, 65, 66]. A flask containing 95% ethanol was perfused with air at a rate of 0.2 to 0.3 l/min to generate ethanol vapor. Ethanol vapor was then combined with a different air stream to give a total flow rate of approximately 4 l/min and delivered to mice in chambers comprised of an airtight top, a vapor inlet, and an exhaust outlet (Allentown Inc., Allentown, NJ). A bout of ethanol vapor exposure consisted of 16 h of ethanol exposure followed by 8 h of withdrawal. This cycle was repeated for four consecutive days. Prior to placement in chambers, mice received intraperitoneal injections of a loading dose of ethanol (20% *v*/*v*, 1.5 g/kg) and pyrazole (68.1 mg/kg for HDID-1 and 34 mg/kg for HS/Npt) in order to achieve BALs of 150–200 mg/dl. Ethanol-naïve mice were handled similarly but were injected with a solution of only pyrazole and placed in a chamber that received only air. Brains were dissected for electrophysiological experiments 24 h after the last ethanol exposure.

### Blood Alcohol Levels

Following each day of vapor or air exposure, tail blood samples were collected immediately upon removal from the vapor chambers and BALs were measured by gas chromatography using a Bruker 430-GC (Bruker Corporation, Fremont, CA) equipped with a flame ionization detector and Combi PAL autosampler. Two, 5-μl samples of blood were collected and added to 10-ml vials containing 45 μl of saturated sodium chloride solution. Samples were warmed to 65 °C, and the solid-phase micro-extraction fiber (SPME; 75 μm CAR/PDMS, fused silica; Supelco, Bellefonte, PA) was used to absorb ethanol vapor from the samples. The stationary phase was a capillary column (30 m × 0.53 mm × 1 μm film thickness; Agilent Technologies, Santa Clara, CA) and helium, at a flow rate of 8.5 ml/min was used in the mobile phase. Ethanol peaks were analyzed using CompassCDS Workstation software (Bruker Corporation, Fremont, CA), and calibration was performed using ethanol standards. Only animals that achieved target BALs of 150–200 mg/dl for the last three consecutive days in the chamber were used for electrophysiological recordings.

### Patch Clamp Electrophysiology

Mice were approximately 10 weeks old at the time of slice preparation [67]. Whole-cell voltage-clamp recordings were made from putative Drd1-MSNs in the AcbSh, and cells expressing CTB were identified by fluorescence microscopy using a BX50 microscope (Olympus) mounted on a vibration isolation table. Recordings were made following previously established procedures [26, 66].

Glutamatergic afferents were stimulated with a stainless steel bipolar stimulating electrode (FHC, Inc., Bowdoin, ME) placed about 150–300 μm from the cell body. Excitatory postsynaptic currents (EPSCs) were acquired using a PC-One amplifier (Dagan Corporation, Minneapolis, MN), filtered at 1 kHz, and digitized at 10–20 kHz with a Digidata 1440A interface board using pClamp 10.2 (Axon Instruments, Union City, CA). Cells were first current clamped to assess resting membrane potential (RMP). Cells with an RMP > − 70 mV were excluded from further analysis as they were not likely representative of an MSN. Cells were then voltage clamped, and EPSCs were evoked by local stimulation every 15 s for at least 10 min. LTD was induced with conditioning stimuli of 500 pulses at 1 Hz, paired with continuous postsynaptic depolarization to − 50 mV. EPSCs were then monitored for 30 min after pairing every 15 s. The magnitude of LTD was calculated by averaging normalized EPSC values from 20 to 30 min after the pairing protocol and comparing to the average normalized EPSCs during the 10-min baseline. Plasticity was determined if the average EPSCs between 20 and 30 min post-pairing was greater than 2 standard deviations away from the 10-min baseline. Data from each neuron within a treatment group were combined and shown as percent baseline values.

### Experimental Design and Statistical Analysis

For the electrophysiological experiments, summary data are shown as mean ± SEM. Statistical significance from baseline within each treatment group was defined as *p* < 0.05 and calculated using a two-tailed Student’s *t* test (assuming equal variance). Group comparisons were calculated using two-way ANOVA and Bonferroni post hoc tests (significance was defined as *p* < 0.05). For gene expression experiments, an empirical Bayes-moderated *t* statistic was used, and statistical significance was defined as *p* < 0.05. Data tables reporting the fold regulation include separate columns for both raw and adjusted *p* values (Table S1 in Online Resource 2). All analyses were conducted in R Studio Version 0.99.903 (running R version 3.2.2) and IPA version number 36601845. Additional information regarding statistical analyses, experimental design and sample sizes (*N*) are provided in the “Materials and Methods” and in the figure and table legends.

## Electronic Supplementary Material

ESM 1 (PDF 640 kb)

ESM 2 (XLSX 40.0 MB)

ESM 3 (XLSX 150 MB)
