# Supplementary material for: Dissecting Brain Networks Underlying Alcohol Binge Drinking Using a Systems Genomics Approach
Source: Mol Neurobiol. 2018 Jul 30;56(4):2791–810. doi: 10.1007/s12035-018-1252-0 (PMC6459809; doi:10.1007/s12035-018-1252-0)
Supplement: Supplementary file 1 — (PDF 640 kb) [file 12035_2018_1252_MOESM1_ESM.pdf]

Online Resource 1
Electronic Supplementary Material (ESM) 1

Dissecting brain networks underlying alcohol binge drinking using a systems genomics approach
Molecular Neurobiology

Laura B. Ferguson[1,2], Lingling Zhang[1], Daniel Kircher[1], Shi Wang[1], R. Dayne Mayfield[1], John C. Crabbe[5,6], Richard A. Morrisett[1], R. Adron Harris[1], Igor Ponomarev*[1]

***Corresponding Author**: Igor Ponomarev, PhD
The Waggoner Center for Alcohol and Addiction Research
The University of Texas at Austin,
Austin, Texas, United States of America
Email: ponomarev@utexas.edu

Fig. 1

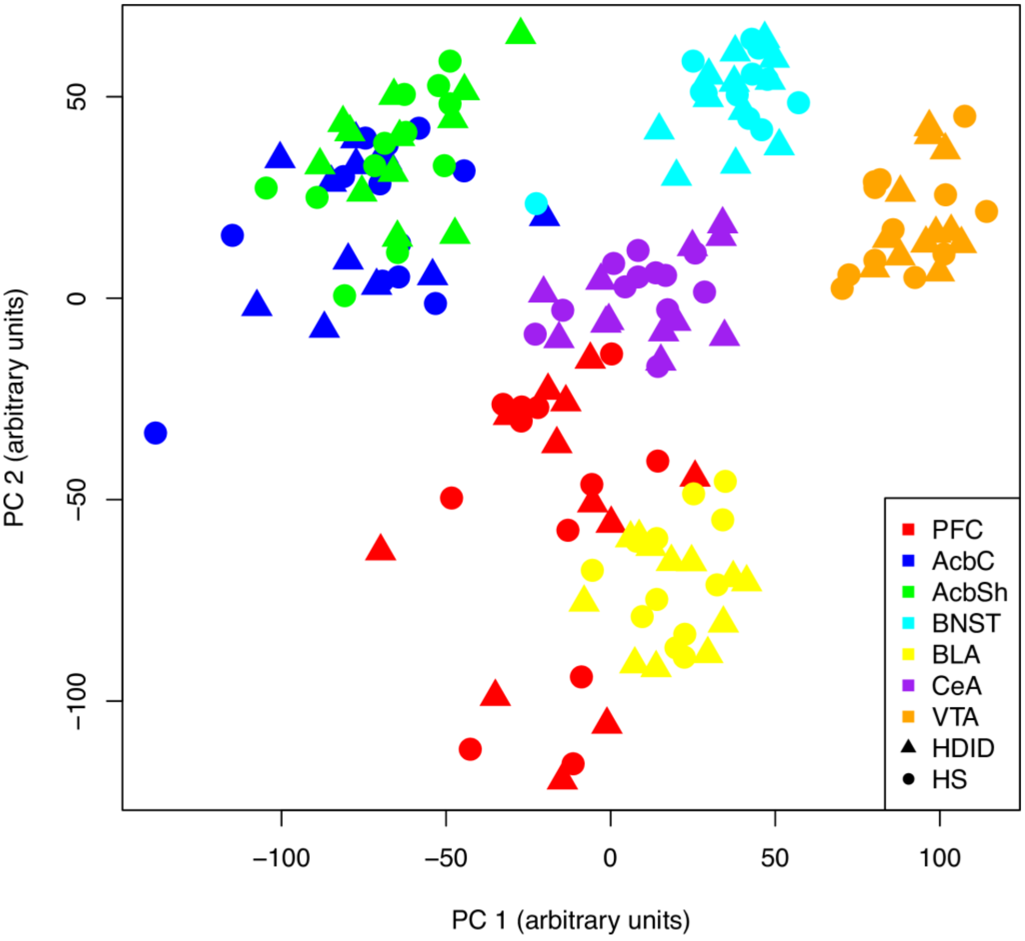

PRINCIPLE COMPONENTS ANALYSIS (PCA) OF THE GENE EXPRESSION DATA.

We performed PCA on the quantile normalized expression data and plotted the first 2 principle components. The first (first 2) principle component accounted for 22.6% (37.5%) of the variance in the data and 30 out of 168 principle components were required to explain 80% of the variance in data

Fig. 2

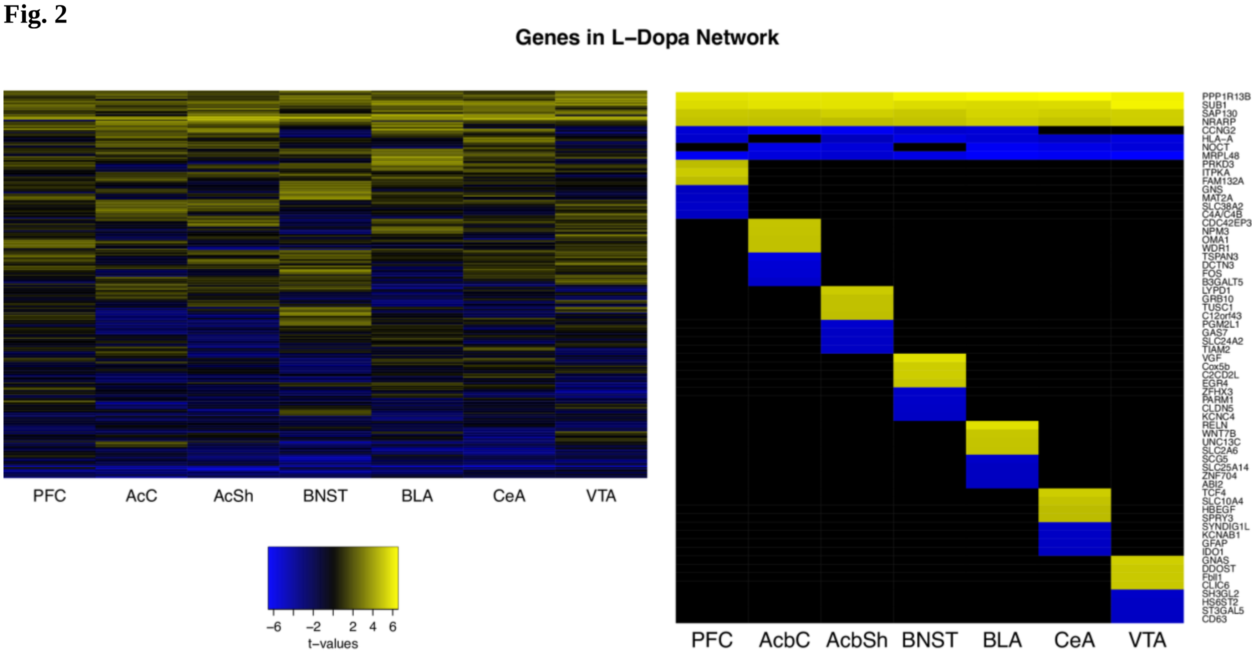

SELECTION-RESPONSIVE GENES IN THE L-DOPA-REGULATED NETWORK.

Ingenuity Pathway Analysis revealed that l-dopa was predicted to be an upstream regulator of the gene expression changes observed in HDID-1 mice (relative to HS/Npt control mice) in each brain region. The heatmap was built in the R programming language and displays the t-values for each gene in the l-dopa network for each brain region analyzed in this study. The rows correspond to genes in the l-dopa-regulated network and columns to brain regions. Blue corresponds to down-regulation and yellow to up-regulation in HDID-1 mice versus HS/Npt control mice. Some of the l-dopa-regulated genes were changed in most brain areas, and some were brain-region specific. Selected genes are displayed (right) from the main categories (from top to bottom): gene up-regulated in all brain regions, genes down-regulated in most brain regions, genes selectively up- or down-regulated in distinct brain regions. PFC – prefrontal cortex, AcbC – accumbens core, AcbSh – accumbens shell, BNST – bed nucleus of the stria terminalis, BLA – basolateral amygdala, CeA – central nucleus of the amygdala, VTA – ventral tegmental area

Fig. 3

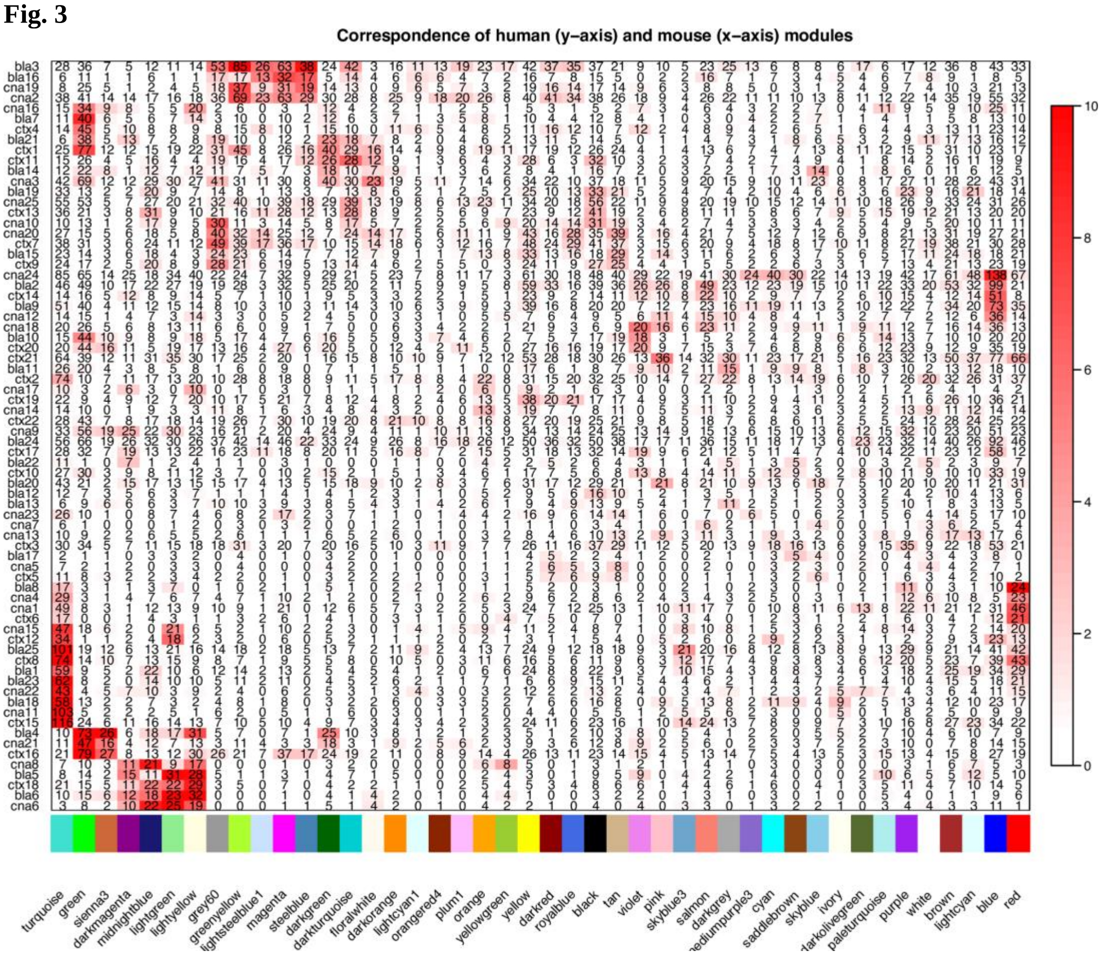

REGULATION OF GENE EXPRESSION PATTERNS IS CONSERVED ACROSS SPECIES.

Gene networks were constructed using the Weighted Gene Correlation Network Analysis method for human (Ponomarev et al., 2012) and mouse brain samples. The gene expression pattern across samples was compared between genes, and genes with similar expression profiles were grouped together into modules. We converted the mouse gene symbols to their corresponding human gene symbol and assessed the overlap of the mouse and human modules using a hypergeometric test. The heatmap plots the –log hypergeometric p-values of the overlap between the mouse (x-axis) and human (y-axis) modules, where values greater than 1.3 are significant at $p < 0.05$. A maximum of 10 was chosen as a ceiling for display purposes. Gene network analyses and figure production performed with the R programming language. Ponomarev I, Wang S, Zhang L, Harris RA, Mayfield RD (2012) Gene coexpression networks in human brain identify epigenetic modifications in alcohol dependence. The Journal of neuroscience : the official journal of the Society for Neuroscience 32 (5):1884-1897. doi:10.1523/JNEUROSCI.3136-11.2012
